# Cost-Effective Offloading of Diabetic Foot Ulcer in a Resource-Crunch Setting: A Case Report

**DOI:** 10.7759/cureus.51173

**Published:** 2023-12-27

**Authors:** Jay Tewari, Shubhajeet Roy, Anadika Rana, Ajoy Tewari

**Affiliations:** 1 Internal Medicine, King George’s Medical University, Lucknow, IND; 2 Faculty of Medicine, King George's Medical University, Lucknow, IND; 3 Internal Medicine, King George's Medical University, Lucknow, IND; 4 Diabetes and Endocrinology, Jai Clinic & Diabetes Care Center, Lucknow, IND

**Keywords:** diabetes mellitus, diabetic foot ulcers, type 2 diabetes, resource-limited setting, offloading, diabetes type 2, diabetic foot ulcers management

## Abstract

Diabetic foot ulcers (DFUs) pose a significant threat to people with diabetes, particularly in regions with limited healthcare resources, such as India. This case report focuses on a cost-effective offloading strategy for managing a chronic non-healing heel ulcer in a 55-year-old female with uncontrolled type 2 diabetes mellitus. While the gold standard for DFU management often involves total contact casts, this method may not be practical for all patients. Our approach involved repurposing used gloves and surgical paper tape for offloading, resulting in quick healing of the ulcer within six weeks. Achieving euglycemic status and sufficient wound debridement were key components of the treatment. This case highlights the importance of resource-efficient strategies in DFU management, especially in settings where traditional methods face practical limitations. Future research is needed to validate the efficacy of such approaches and pave the way for more accessible and effective treatments for DFUs.

## Introduction

Diabetes mellitus has attained a pervasive global status, with India emerging as a prominent hub for its prevalence and associated complications. According to a recent study published by Anjana et al., India has been found to have more than 101 million people living with diabetes compared to 70 million people in 2019, an increase of 44% in the last four years [1]. Among these, diabetic foot complications are a significant contributor to diminished quality of life. It has a bearing on India's predominantly youthful population, and associated morbidity contributes to loss of livelihood and financial consequences. A noteworthy consequence is the alarming statistic that 20% of diabetic foot infections ultimately end with the unfortunate necessity of amputation [2]. The four pillars of diabetic foot ulcer (DFU) management are debridement, offloading, maintaining perfusion, and good diabetes control. According to the International Working Group on the Diabetic Foot guidelines, the management of DFUs often involves implementing an "offloading" strategy to alleviate pressure [3]. Offloading in diabetic foot management means relieving pressure from an ulcerated area. In other words, it means the redistribution or removal of detrimental forces applied to the foot [4]. The specific type of off-loading technique used depends on the location of the wound, and other factors like ischemia and infection [5]. These guidelines suggest that the primary approach for promoting wound healing in DFU is the use of a non-removable, knee-high off-loading device like a total contact cast [5]. However, individuals with lower limb edema, increased limb size, leg ulcers, and eczema-like skin conditions, may encounter difficulties in using knee-high off-loading interventions, potentially leading to compromised outcomes [6]. Moreover, total contact cast is not practical and time-consuming and needs expertise to implement. The heel bears the entire weight of the body thus heel ulcers are difficult to heal. We present a case of a chronic non-healing foot ulcer of the heel that was managed by a cost-effective offloading method, which can be easily done in a busy medical practice.

## Case presentation

**Figure 1:**
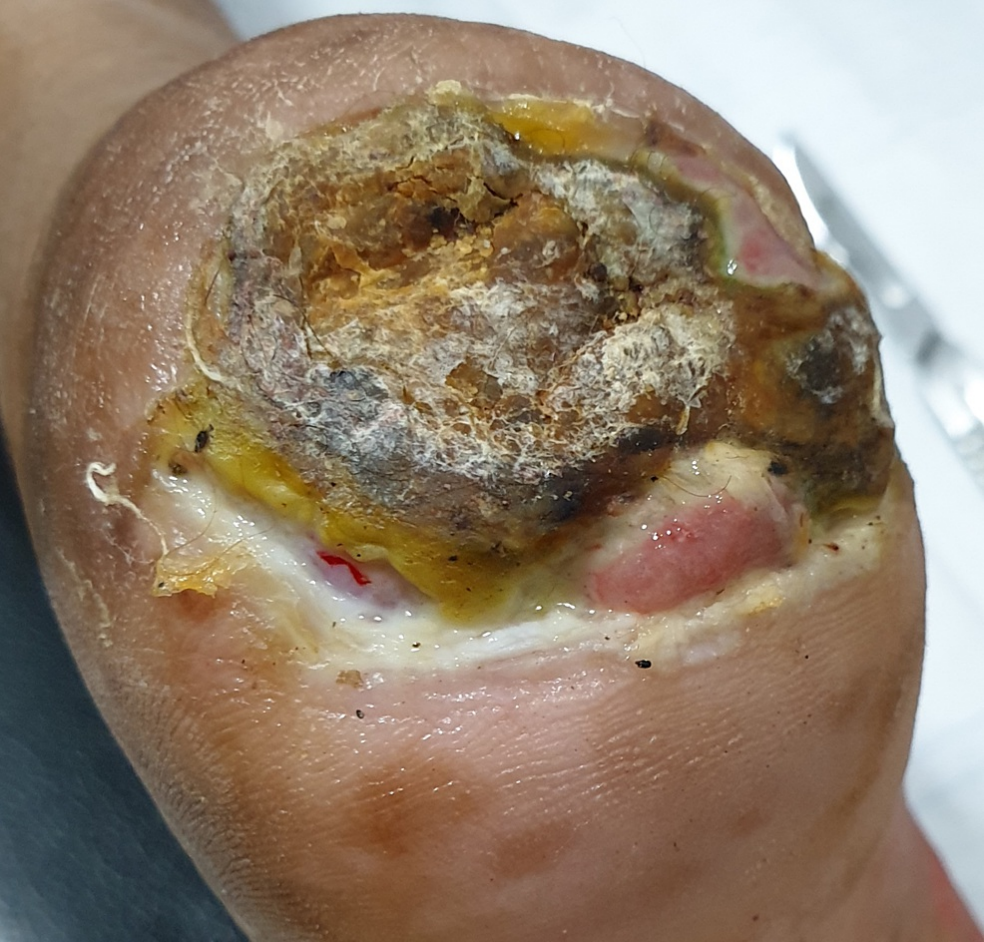
A chronic non-healing ulcer on the plantar surface of the heel.

A 55-year-old female presented to the internal medicine outpatient department with a chronic non-healing ulcer on the plantar surface of the heel, as shown in Figure 1.

There was a history of uncontrolled type 2 diabetes mellitus for the last 15 years and the presence of a non-healing ulcer for the last four months. Initially, she presented with blood glucose in the range of 250-300 mg%. Her sister was diabetic, but her parents’ status was not known. She was on medications, prescribed four years back, by some local healthcare practitioner, but she was irregular in taking her medications, and neither performed regular self-monitoring of glucose. Her total leukocyte count was 14000/cu.mm, with neutrophil being 80%. She was educated about self-monitoring of glucose and was asked to come with her four-point testing every third day at the time of dressing. Insulin was added to the known regimen of oral hypoglycemic agents and the dose was uptitrated as per the sugar log to achieve euglycemic status.

A three-minute foot examination was performed, which involved history, physical examination, and relevant education [7]. On examination, there was an epithelialized non-healing chronic ulcer on the plantar surface of the heel, four by five centimeters in size, probing was done post-debridement, and the depth extended to subcutaneous tissue and underlying fascia. The posterior tibial and dorsalis pedis pulses were palpable. Ankle reflexes were normal, the skin was dry and the shins revealed bilateral diabetic dermopathy, the interweb spaces were clean, the nails had no deformity, and there were no bony deformities. The footwear revealed signs of high pressure on the heel bilaterally. After examination, a brief oral and written communication was given about foot care and hygiene.

**Figure 2:**
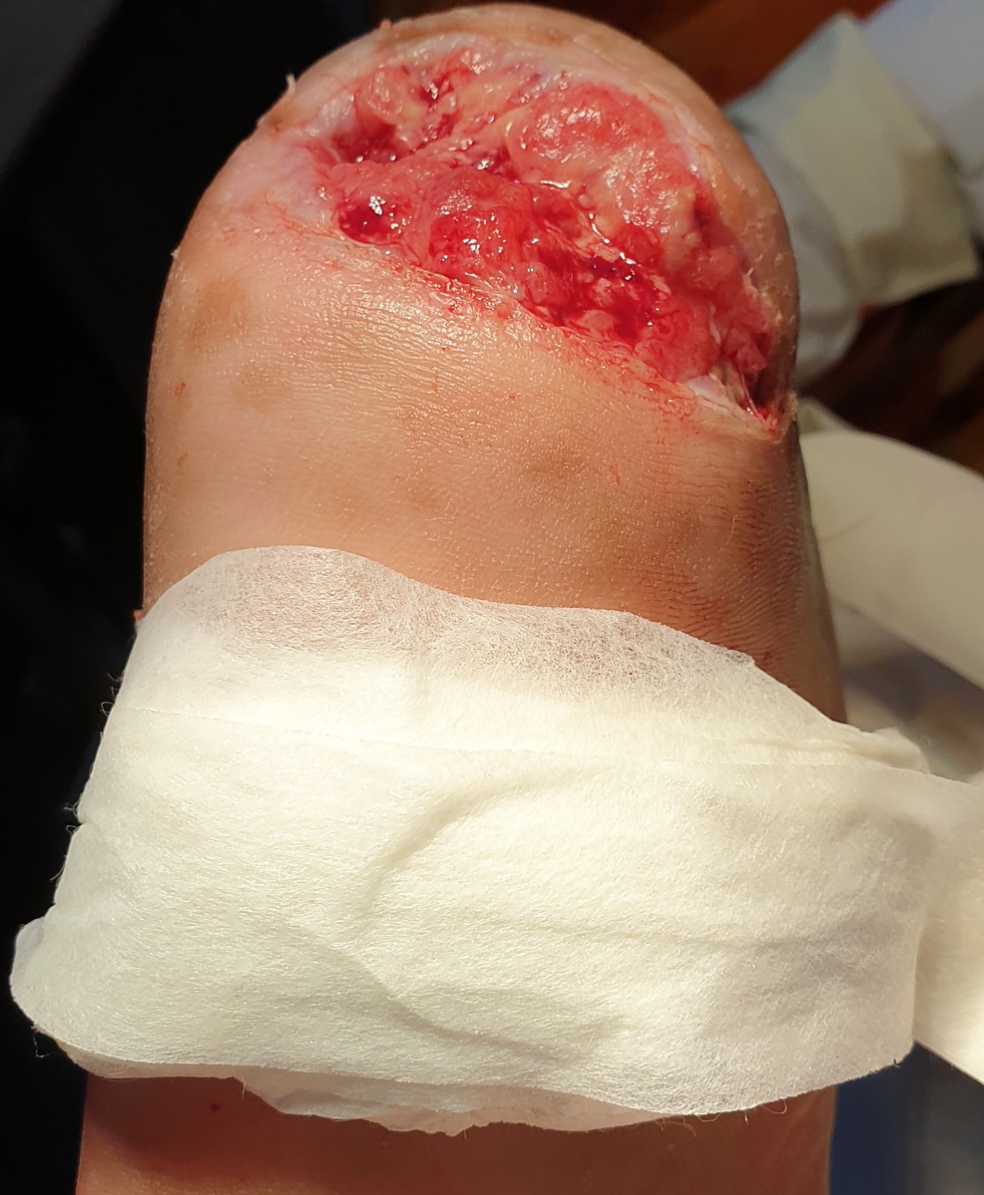
Offloading technique: used gloves rolled inside out; edges rounded and encapsulated with surgical paper tape and secured with surgical paper tape distal to the heel lesion.

It was managed by first achieving euglycemic status with a combination of injection human mixtard 30/70 (mixture of 30% regular and 70% neutral protamine Hagedorn) that was added to a combination of glimepiride 2 mg and metformin 500 mg twice a day and was uptitrated to a dose of 30U in the morning before breakfast and 14U before dinner to achieve euglycemic status. A combination of amoxicillin 500 milligrams and clavulanic acid 175 milligrams was administered per oral once a day for two weeks to prevent any surgical site infections. Extensive debridement was done on the initial visit. The offloading technique used was as follows: used gloves were rolled inside out; the edges were rounded and encapsulated with surgical paper tape. After debridement of the wound, the offloading system was secured with surgical paper tape distal to the heel lesion, as in Figure 2.

**Figure 3:**
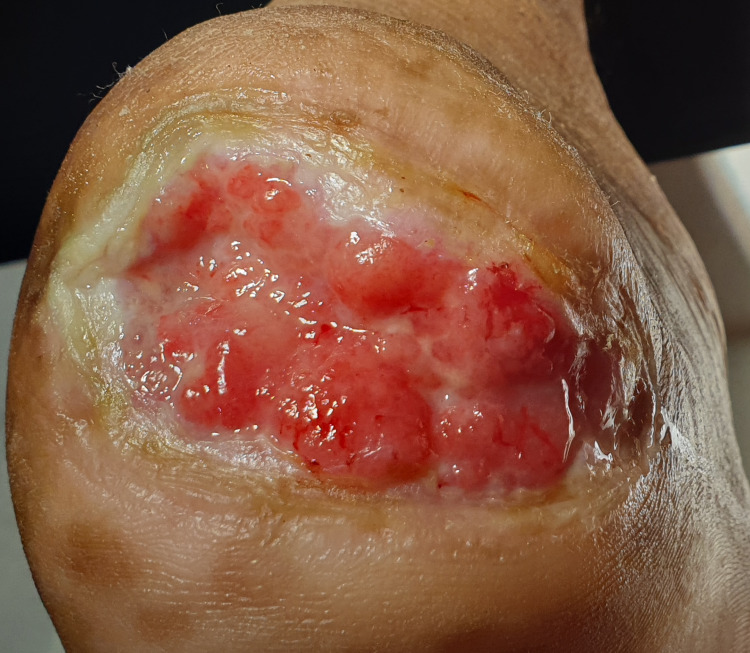
Healed ulcer after six weeks.

Dressing (a sterile surgical pad manufactured by Johnson & Johnson (New Brunswick, NJ) was used for surgical dressings, and temporary offloading with surgical gloves was done during the period of intervention to promote healing) and debridement were repeated every third day with normal saline to prevent biofilm formation, followed by a change of the distal offloading. This simple technique resulted in the healing of a chronic ulcer in six weeks, as shown in Figure 3.

The patient was ambulatory all along, so no extra precautions associated with prolonged immobilization were needed. The patient was given offloading footwear with proximal offloading to prevent recurrence after the complete epithelialization of the wound with advice for follow-up and regular self-monitoring of blood sugar at home.

## Discussion

DFUs constitute a distressing facet of diabetes progression, impacting approximately 15% of individuals with diabetes. A comprehensive systematic review has demonstrated a reduction in the amputation rate among diabetic foot patients by employing timely and effective debridement [8]. The pursuit of euglycemic status as a therapeutic approach is supported by evidence indicating that glycemic control exerts a delaying influence on the onset and progression of diabetic retinopathy, nephropathy, and neuropathy in patients with insulin-dependent diabetes mellitus [9,10]. Debridement, involving the meticulous removal of necrotic tissue, peri-wound callus, and foreign matter to reveal viable tissue, is the foundation of ulcer management. Proper debridement is essential for curbing infection risk and mitigating peri-wound pressure, which has the potential to impede normal wound contraction and healing [11].

Despite optimum glucose control, debridement, and the use of antibiotics, most DFUs do not heal due to a lack of proper offloading. Particularly challenging are heel ulcers, characterized by their enduring burden of supporting the entire body weight. Cast devices used for DFU include total contact casts, below-knee walking casts, Scotchcast™/fiber glass/polyester resin boots, removable cast walkers/instant total contact casts, and removable heel casts. Non-cast devices include half-shoes (healing sandals), crutches, and wheelchairs [4]. While total contact casting is upheld as the gold standard based on multiple conducted studies, it carries inherent limitations. Challenges encompass the potential emergence of new ulcers, constraints on daily wound care, compromised mobility, potential financial burdens, and the requirement for specialized personnel [12,13]. Particularly within resource-constrained nations, burdened by diabetes prevalence, such as India, the feasibility of this approach is questionable. Consequently, novel therapeutic modalities are imperative to address these challenges. One such innovative approach, as exemplified in the present case, involves repurposing pre-used gloves to minimize treatment costs. This strategy offers promise as a potential solution for enhancing DFU management, particularly in settings where resource limitations are a crucial consideration. This method was a modification of the Mandakini system of offloading developed by Sunil V. Kari from India [14]. We used surgical paper tape instead of Dynaplast. The offloading system was changed every third day to obviate the chance of infection, maceration, and loss of recoil of the offloading system.

## Conclusions

While traditional methods of treatment are effective, they bring forth their own set of challenges that limit their application in resource-limited scenarios. Hence, the exploration of cost-effective methods, such as the one used here, provides promise in addressing the challenges faced. Future research endeavors are required to ascertain the true impact of repurposing used gloves in the management of DFUs. Also, further innovations are required in this space to pave the way for a more effective and accessible treatment for DFUs.
